# Exploring Parkinson’s Disease-Associated Depression: Role of Inflammation on the Noradrenergic and Serotonergic Pathways

**DOI:** 10.3390/brainsci14010100

**Published:** 2024-01-20

**Authors:** Tuane Bazanella Sampaio, Marissa Giovanna Schamne, Jean Rodrigo Santos, Marcelo Machado Ferro, Edmar Miyoshi, Rui Daniel Prediger

**Affiliations:** 1 Department of Pharmacy, State University of Centro Oeste, Guarapuava 85040-167, PR, Brazil; 2 Graduate Program in Biomedical Sciences, Department of Pharmaceutical Sciences, State University of Ponta Grossa, Ponta Grossa 84030-900, PR, Brazil; 3 Graduate Program in Biomedical Sciences, Department of General Biology, State University of Ponta Grossa, Ponta Grossa 84030-900, PR, Brazil; 4 Graduate Program in Pharmacology, Department of Pharmacology, Federal University of Santa Catarina, Florianópolis 88040-900, SC, Brazil

**Keywords:** Parkinson’s disease, neuroinflammation, antidepressants, locus coeruleus, raphe nuclei

## Abstract

Parkinson’s disease (PD) is a multifactorial disease, with genetic and environmental factors contributing to the disease onset. Classically, PD is a movement disorder characterized by the loss of dopaminergic neurons in the nigrostriatal pathway and intraneuronal aggregates mainly constituted of the protein α-synuclein. However, PD patients also display non-motor symptoms, including depression, which have been linked to functional abnormalities of non-dopaminergic neurons, including serotonergic and noradrenergic ones. Thus, through this comprehensive literature review, we shed light on the noradrenergic and serotonergic impairment linked to depression in PD, focusing on the putative involvement of inflammatory mechanisms.

## 1. Introduction

Parkinson’s disease (PD) is the second most common neurodegenerative disease and the most frequent movement disorder. Epidemiological studies evaluating PD incidence, prevalence, ethnicity, sex ratio, and risk factors have been performed worldwide, but the results are inconsistent due to methodological differences and variability in diagnostic criteria [1,2]. PD incidence ranges from 10 to 18 per 100,000 person-years, with the prevalence significantly increasing with age. Although aging is the most significant risk factor for PD development, being male and having Hispanic ethnic origin increases the risk for the disease. Also, there is a higher PD prevalence in Europe, North America, and South America compared with that in African, Asian, and Arabic countries [2].

However, there is a consensus that PD etiology is a multifactorial disorder involving both genetic and environmental factors. Most cases are sporadic and idiopathic, with only 10% linked to genetic causes. Environmental factors that increase PD risk include pesticide exposure, head injuries, rural living, agricultural occupation, and well-water drinking. On the other hand, genetic risk factors are considered when linked to PD family history or tremor, which include *LRRK2*, *GBA*, and *SCNA* gene mutations [2]. These factors can be associated with central and peripheral inflammation, contributing to the onset and progression of PD [1,2].

PD pathophysiology is classically characterized by the loss of dopaminergic neurons in the substantia nigra (SN) and the presence of intraneuronal aggregates mainly constituted of the protein α-synuclein, denominated Lewy neurites, and Lewy bodies. PD motor symptoms are primarily attributed to a decrease in striatal dopamine levels associated with dopaminergic neurodegeneration in the SN. Of note, motor symptoms only manifest when 60–70% of the nigrostriatal pathway dopaminergic neurons have already been lost and the dopamine content in the striatum is reduced by about 70–80% [2]. However, it is known that there is widespread involvement of other brain areas and peripheral tissues [3].

In this sense, the classical view of PD as a primary movement disorder characterized by cardinal motor signs (tremor, rigidity, bradykinesia, and postural instability) has been drastically altered over recent years, and non-motor symptoms are recognized as relevant clinical manifestations of PD. These non-motor symptoms include cognitive impairment, psychiatric symptoms, autonomic dysfunction, sleep disorders, olfactory dysfunction, pain, orthostatic hypotension, and fatigue. Importantly, non-motor symptoms have been linked to the functional abnormalities of non-dopaminergic pathways, including serotonergic, noradrenergic, and cholinergic neurons, which can be observed even during early pre-motor stages [1,2,3,4].

Considering this, our study aims—through a comprehensive literature review—to shed light on the noradrenergic and serotonergic damage linked to depression in PD, focusing on the putative involvement of inflammatory mechanisms.

## 2. The Interplay of Noradrenergic and Serotonergic Systems in Parkinson’s Disease

Evidence shows that the neurodegenerative process in PD begins many years before the onset of motor symptoms, affecting other central nervous system (CNS) structures beyond the nigrostriatal pathway. Indeed, Braak and colleagues used Lewy neurites and bodies to stage PD, demonstrating that neuronal changes in PD began in lower brainstem regions, such as the medulla and pons, and then progressed to the mesencephalon [3]. Although Lewy body formation is not entirely understood, as well as the exact mechanisms of PD progression, they found that the dorsal motor nucleus of the vagus nerve (cholinergic neurons), raphe nuclei (RNs—serotonergic neurons), and coeruleus–subcoeruleus complex (noradrenergic neurons) were previously affected (stages 1 and 2) by a α-synuclein pathology of the midbrain area (dopaminergic neurons) (stage 3) [3]. Moreover, GABAergic and glutamatergic changes have been described in PD. Overall, PD seems to be a multisystemic disease in which other neurotransmitter systems beyond the dopaminergic could be responsible for the onset and progression of non-motor symptoms [3,4].

As knowledge about PD advances, post-mortem, imaging, and animal research have reinforced the hypothesis that noradrenergic dysfunction plays a crucial role in the pathogenesis of PD [5,6,7,8]. The locus coeruleus (LC), situated bilaterally in the dorsal pons, is a main source of noradrenaline in the CNS. It contains about 15,000 neurons per hemisphere projected to much of the brain. Due to its neuroanatomical features, the LC modulates several functions, such as homeostasis, cognition, arousal, motor behavior, and sensory processing, implicating that its dysfunction can be associated with a wide range of signs and symptoms [9,10]. 

Corroborating this view, LC degeneration is a neuropathological marker that crosslinks the two more common neurodegenerative diseases: PD and Alzheimer’s disease [8,11]. LC impairments in PD have been demonstrated to occur earlier and in greater magnitude than those in the SN [3,7]. It is estimated that approximately 60% of PD patients show LC degeneration, which ranges from 21 to 93% of neuronal loss [10]. Additionally, neuromelanin-sensitive magnetic resonance imaging (MRI) demonstrated reduced signal intensity in the LC in PD patients, negatively correlating to non-motor symptoms [7]. Moreover, LC loss occurs throughout the nucleus, reaching the peri-LC subcoeruleus area, and the remaining neurons display a reduced size and modified phenotype [8]. 

Associated with LC neuronal loss, there is the depletion of noradrenergic inputs in several cerebral structures, such as the frontal cortex, cerebellum, striatum, hippocampus, and thalamus. In this sense, the resultant noradrenergic dysfunction could be linked to orthostatic and postprandial hypotension, the disruption of the circadian rhythm and arousal/wakefulness cycles, as well as cognitive impairment and psychiatric symptoms [9]. Also, the freezing of gait is linked to noradrenaline levels in the cerebrospinal fluid, being enhanced by treatment with a noradrenaline precursor [12].

Animal studies involving LC degeneration also support noradrenergic dysfunction in PD. Using 6-hydroxydopamine (6-OHDA), a well-characterized catecholaminergic neurotoxin [13], rodents subjected to the LC lesion showed behavioral changes similar to those observed at different stages of PD, such as the disruption of olfactory, cognitive, emotional, and motor functions [5,6,14]. Motor symptoms are also observed when dopaminergic damage caused by 1-methyl-4-phenyl-1,2,3,6-tetrahydropyridine (MPTP), a neurotoxin that induces Parkinsonism in humans [15], or 6-OHDA, are related to LC damage [16,17]. In addition, exogenous intranasal noradrenaline administration was protective against non-motor behavioral impairments induced by 6-OHDA [5]. In contrast, in a genetic model of PD, dopaminergic and noradrenergic damages were not potentiated by SN and LC lesions [18]. Furthermore, the LC, directly and indirectly, controls the catecholaminergic levels. For example, LC stimulation increases the extracellular concentrations of noradrenaline that modulate dopamine release by the ventral tegmental area and SN. Similarly, the RN is stimulated by noradrenaline release [19].

In this way, a growing body of evidence has also drawn attention to the presence of serotonergic dysfunction linked to PD progression [3,20,21]. Serotonin (5-HT) is a monoaminergic neurotransmitter synthesized by neurons projecting from the dorsal raphe (DRN) and median raphe (MRN) nuclei to a significant number of brain structures, including the dopaminergic limbic system. It acts by activating 5-HT receptors, a family with 14 identified subtypes, and it is a crucial behavior modulator with actions on depressive, apathetic, and anxious states [21]. 

According to Braak et al. [3], Lewy body inclusions also can be detected first in serotonergic neurons, then in dopaminergic ones. A depleted serotonergic function has been reported in several studies with PD patients, and various mechanisms have been hypothesized as responsible for the reduction in 5-HT levels. Positron emission tomography (PET) imaging studies using [^11^C]-DASB, a 5-HT transporter (SERT) ligand, indicated that PD patients’ brains present increased SERT binding in the basal nuclei and limbic system regions, suggesting an excessive 5-HT reuptake. Contrarily, the caudate, thalamus, hypothalamus, and anterior cingulate cortex were SERT-depleted in early PD patients, while reduced binding in the putamen, insula, as well as orbitofrontal, posterior cingulate, and prefrontal cortices appeared in advanced PD patients [20]. In addition, resting-state functional MRI revealed a dysconnectivity profile between the DRN and MRN in PD carriers. The DRN was poorly connected with the bilateral prefrontal and cingulate cortices, being correlated with depression, drowsiness, and anxiety. In contrast, the MRN displayed decreased connectivity with the pons, which could affect depressive, cognitive, sleep disturbance, and nociceptive symptoms [21].

Research with animal models of PD induced by nigrostriatal lesions also provoked 5-HT dysfunction. Marked depletion in the striatal and hippocampal 5-HT levels was described in nigral 6-OHDA-lesioned rats [22]. It is interesting to observe that the 6-OHDA, which is supposed to be specifically toxic to catecholamine neurons, was infused into dopaminergic neuron bodies in the SN and caused 5-HT depletion in other brain structures, implying a putative dopaminergic regulation on 5-HT release. In MPTP-lesioned mice, Ishii [23] demonstrated that SN pars compacta degeneration reduced the inhibitory action of dopamine on SN reticulata GABAergic neurons, increasing their inhibitory activity on the MRN, which affected behavior and memory by reducing 5-HT secretion on the hippocampus.

## 3. Inflammation in Parkinson’s Disease

Further to α-synuclein aggregation, other factors are commonly found in PD pathophysiology, such as mitochondrial dysfunction, oxidative and endoplasmic reticulum stress, and inflammation. There is increasing evidence indicating an inflammatory role in PD onset and progression. In turn, oxidative stress is the primary cause of several diseases, especially aging-related ones. Thus, although it remains unclear if there is a majority process, it is essential to highlight that neuroinflammation and oxidative stress exacerbate each other [24].

Neuroinflammation is one of the most critical complications of PD linked to protein aggregates, oxidative stress, neurodegeneration itself, and peripheral immune and inflammatory changes. Although the cause–effect mechanisms remain unclear, neuronal loss in PD is undoubtedly associated with chronic inflammation, which is mediated mainly by microglia and, to a lesser extent, by astrocytes and oligodendrocytes [24,25]. 

Reactive microglia secrete many pro-inflammatory cytokines such as interferon-γ (IFN-γ), tumor necrosis factor-α (TNF-α), interleukin 1-β (IL-1β), IL-6, and upregulates enzymes such as inducible nitric oxide synthase (iNOS) and cyclooxygenase 1 and 2 (COX-1/COX-2) [25,26]. Notwithstanding, activated microglia in PD have been associated with different PD-related genes/proteins like *SNCA* and *LRRK2*. α-synuclein expression changes active immune cells and inflammatory pathways in PD models and humans. Once in the extracellular space, α-synuclein aggregates bind to tool-like receptors (TLRs), inducing robust microglial activation. In addition, fibrillar α-synuclein can activate the NLRP3 inflammasome, increasing levels of IL-1β and caspase-1. Also, α-synucleins accumulate in astrocytes, leading to Lewy body formation and pro-inflammatory cytokine release via the TLR4 pathway [27].

Post-mortem analyses of PD brains identified a significant increase in positive microglia in the SN, putamen, hippocampus, transentorhinal cortex, cingulate cortex, and temporal cortex [28]. In addition, microglial activation has also been found in the olfactory bulb of sporadic and familial PD patients [29]. To access neuroinflammation in vivo is viable using a PET translocation protein (TSPO) tracer, which is concentrated in activated microglia. In PD patients, an increased binding of TSPO was demonstrated in the pons, SN, basal ganglia, and frontotemporal cortex. However, the microglial activation did not present a correlation with the severity of motor symptoms, duration of the disease, and striatal dopamine uptake [30,31]. Such in vivo evidence suggests that microglial activation occurs in early PD stages, remaining relatively stable during disease progression. This may implicate its role in driving the disease through cytokine release.

Although the hypothesis has not been tested, the activated microglia found in the pons [30] could indicate an early dysfunction in the non-dopaminergic pathways. In this sense, patients diagnosed with idiopathic rapid eye movement sleep behavior disorder—an early and non-motor symptom of PD—displayed activated microglia in the occipital lobe and thalamic monoaminergic impairment, which may be linked to project neuronal dysfunctions in the DRN and LC [32].

Noradrenaline is considered an anti-inflammatory and neuroprotective neurotransmitter. Its anti-inflammatory effect has been mainly linked to reduced NF-κB activity by activating β-adrenergic receptors. In astrocytes and microglia, noradrenaline inhibits the NF-κB pathway by increasing the cytosolic expression of the inhibitory IκBα and heat-shock protein 70 (HSP70) [33,34,35]. Additionally, peroxisome proliferator-activated receptor gamma (PPARγ) expression is stimulated by noradrenaline in astrocytes and neurons, stabilizing their resting status [36]. As a consequence, noradrenaline induces a reduction in TNF-α, IL-6, IL-8, iNOS, COX-2, and monocyte chemoattractant protein-1 (MCP-1) levels, as well as rapid IL-10 secretion (an anti-inflammatory cytokine) in response to multiple TLR signals [37,38]. Moreover, Mittal and colleagues [39] found that the treatments with β2-adrenergic receptor ligands modulate α-synuclein expression, suggesting that β2-adrenergic receptor agonists reduce the risk of developing PD by countering the α-synuclein-induced inflammatory pathways.

In its turn, 5-HT reuptake is stimulated by pro-inflammatory cytokines, such as TNF-α and IL-1β. TNF-α increases SERT activity and expression in glioma cell lines and primary astrocytes [40], while IL-1β stimulates SERT activity both in RN cell lines in vitro and synaptosomes in the frontal cortex, hippocampus, striatum, and midbrain of mice [41]. Another possible mechanism for weaker serotonergic activity is the cytokines’ stimulating effect on the enzyme indoleamine 2,3-dioxygenase, causing a depletion in tryptophan and consequent impairment in 5-HT synthesis [42].

Moreover, dopamine is a crucial neurotransmitter for modulating immune cell functions. Whereas dopamine receptor type 1 (D1R) and 2 (D2R) activation in microglia and astrocytes seems to have an anti-inflammatory effect, dopamine receptor type 3 (D3R) deficiency/inhibition is involved in the increased expression of TNF-α, IL-1β, and iNOS. On the other hand, peripheral D1R activation was associated with increased nuclear transcription factor nuclear factor kappa b (NF-κB) and NLRP3 inflammasome, with a consequent increase in IL-6, IL-1β, and IL-18 levels. These controversial data could be related to species variation in immune-related pathways, dopamine receptor expression, as well as differences in used dopamine concentrations [25].

In this sense, active microglia can release reactive oxygen and nitrogen species (ROS/RNS) in response to environmental toxins and endogenous stimuli, increasing carbonyl and nitration protein modifications, lipid peroxidation, DNA damage, and glutathione (GSH) depletion. Lewy bodies display increased levels of oxidized and nitrated proteins, suggesting the vital role of oxidative stress in PD neurotoxicity. Most of the neurodegeneration in PD is due to the mitochondrial chain’s complex I deficiencies, which increase the ROS and reduces ATP production, leading to impairments of intracellular components and consequent death [24].

Indeed, reduced complex I activity has been described in the sporadic PD’s SN in both early and advanced stages [43]. Environmental factors, aging, dopamine metabolism, reactive iron stored as neuromelanin, and genetic mutations in PD-associated proteins contribute to mitochondrial dysfunction, which precedes ROS formation. It is known that dopamine metabolism, from its synthesis until degradation, involves enzymes that produce H_2_O_2_ as a product of their activities. Similarly, the high concentration of iron, commonly found in the SN of PD carriers, induces metal binding in low-affinity sites, in which iron accumulates in its reactive form and catalyzes with Fenton’s reaction [24,44].

Once oxidative stress is characterized by an imbalance between ROS/RNS production and the antioxidant system, oxidative damage is associated with a GSH deficit. GSH is a major thiol antioxidant and redox buffer that scavenges a wide range of ROS, regenerates other antioxidants such as vitamins C and E, and is a substrate for several antioxidant enzymes. Importantly, reduced GSH levels in the SN seem to precede mitochondrial dysfunction and dopamine decrease [44]. Moreover, nicotinamide adenine dinucleotide phosphate (NADPH) oxidase (NOX), an extensive producer of extracellular ROS, is upregulated in PD, and its expression coincides with activated microglia, increasing the pro-inflammatory response [24,44].

## 4. The Putative Interconnected Role of the Noradrenergic and Serotonergic Systems on Depression Associated with Parkinson’s Disease

Depression is a disorder of high social impact, as it generates high treatment costs, involving a more significant use of health services and low productivity at work. Depressed individuals are more susceptible to various comorbidities, such as coronary artery disease, hypertension, stroke, and type 2 diabetes mellitus. On the other hand, depression can be considered a risk factor for the development of neurodegenerative diseases, including PD [1]. Although depression is a common symptom of other chronic conditions and often occurs in the elderly population, evidence indicates that depression is more frequent in PD patients than in individuals of the same age group or patients with other comorbidities. In fact, among the non-motor symptoms of PD, depression is the psychiatric disorder most frequently associated with PD and the one that most impacts the daily activities and quality of life of PD patients [45,46].

The loss of energy and interest, anhedonia, difficulty in making decisions, feelings of sadness, helplessness, and rapid eye movement (REM) sleep disorder are some of the symptoms resulting from depression most observed. It is common for PD patients to become passive or withdrawn, to have a loss of appetite, fatigue, sleep disorders, and slow cognitive processes, and to slowly respond to questions [4]. This creates an overlap of symptoms between depression and PD, which frequently are confused and neglected, impairing the treatment of the individual and accelerating the cognitive decline and worsening of quality of life. Even when diagnosed, it is estimated that only 25% of patients receive effective antidepressant treatment [1,46].

Approximately 25% of PD patients suffer from depression before manifesting motor symptoms, and 40–50% of PD carriers will display depressive symptoms throughout the illness [1]. Depression may precede the PD diagnosis by up to 20 years, but its incidence increases during the 3–6 years preceding the diagnosis of PD [4,45]. Previous studies have shown that depressed patients and the prior use of antidepressants increase the probability of developing PD [46].

It has been pointed out that the neurobiology and clinical manifestation of depression in PD can differ from those observed in the general population. Unlike major depression, individuals with PD have more dysphoria and irritability, lower feelings of guilt, and incidences of suicide [1]. One of the clues to the understanding of this difference lies in the lack of responsiveness to the classic treatment with antidepressants; 50% of patients are refractory to classical antidepressants. In clinical terms, PD-associated depression has obtained better therapeutic indices when treated with pramipexol, a dopaminergic agonist, and nortriptyline, a tricyclic antidepressant [46]. In this way, although dopaminergic neurotransmission is seen as one of the possible causes of PD-associated depression, other neurotransmitters are also reduced, for example, 5-HT and noradrenaline [1,3] (Table 1).

Considering the depression pathophysiology, one of the hypotheses most accepted is the monoaminergic one linked to a reduction in the monoamine (dopamine, 5-HT, and noradrenaline) levels [1]. The intensity of serotonergic damage has been associated with depressive behavior in PD patients. Transcranial sonography revealed a reduced RN echogenicity, which is more prominent in depressed PD patients than in non-depressed ones [48]. Recently, apathy, depression, and anxiety in PD patients were positively correlated with the intensity of serotonergic lesions in the nucleus accumbens [53]. Also, depressed PD patients presented a decreased postsynaptic 5-HT_1A_ receptor density in the limbic structures, detected with PET with [^18^F]MPPF [51], and reduced serotonergic metabolite 5-hydroxyindoleacetic acid (5-HIAA) in the cerebrospinal fluid (CSF) [51]. In this way, subthalamic nucleus deep (STN) brain stimulation (DBS) relieves motor dysfunction but can also worsen psychiatric behaviors in PD patients, such as depression and impulsivity [54]. This effect may be linked to a 5-HT dysfunction in PD patients since STN-DBS induces a sustained inhibition of the DRN and, consequently, the serotonergic inputs in the hippocampus, prefrontal cortex, and MRN in rodents [55,56].

In contrast, a large single photon emission computed tomography (SPECT) study using ^123^I-FP-CIT, a SERT and dopamine transporter (DAT) binder, found no association between serotonergic lesions in the RN and depression scores in early-stage PD patients [52]. Also, Frisina et al. [50] reported no post-mortem neuropathological differences between depressed and non-depressed PD patients at the DRN, amygdala, and cortical regions. According to the above-mentioned, since depressed PD patients displayed increased SERT binding [20], the depressive symptoms may not have been associated with serotonergic neurodegeneration but were related to an increased 5-HT reuptake or 5-HT synthesis deficits.

Furthermore, depression has been widely linked to inflammatory processes. Once noradrenaline is considered an anti-inflammatory molecule and LC damage results in noradrenergic dysfunction, noradrenaline may be a key neurotransmitter in PD-associated depression [57]. Importantly, LC impairment precedes midbrain damage and motor symptom development in PD, supporting its correlation with some of the early non-motor symptoms, including depression [3,7]. On the other hand, LC impairment is also observed in neuropsychiatric diseases, such as depression [49,57]. Using an in vivo marker for dopamine and noradrenaline transporter binding–[^11^C]RTI-32 PET, Remy et al. [49] demonstrated that depressed PD patients had reduced [^11^C]RTI-32 binding in the LC and limbic areas, such as the amygdala, anterior cingulate cortex, ventral striatum, and thalamus, indicating noradrenaline loss in these structures.

Moreover, depressed PD patients do not respond consistently to usually prescribed antidepressants. Selective 5-HT reuptake inhibitors (SSRIs) are the first-line antidepressant drugs. Although most studies suggest that SSRIs are effective in reducing depressive symptoms in PD, their efficacy seems to be lesser than dual 5-HT and noradrenaline reuptake inhibitors (SNRIs) or tricyclic antidepressants (TCAs) [58]. TCAs, noradrenaline and 5-HT reuptake inhibitors, such as desipramine and nortriptyline demonstrated clinic superiority for PD-associated depression over, respectively, the SSRIs citalopram and paroxetine [59,60]. In turn, paroxetine data are controversial [60,61]. Citalopram improved response inhibition and increased prefrontal activation in patients with moderate-to-severe PD [62]. In this way, the SNRIs venlafaxine and duloxetine significantly improved depression in PD patients in a similar manner to SSRIs [61,63]. In addition, 5-HT_2C_ antagonists are used to treat both PD and depression, and its mechanism of action seems to be related to dopamine release modulation in distinct brain regions [64].

On the other hand, it has also been suggested that depressive symptoms shift during PD evolution, with more dopamine-related symptoms, like anhedonia, arising in advanced stages [65]. In this sense, dopaminergic agonists such as pramipexole, ropinirole, rotigotine, and piribedil can be useful. Their indications are associated with PD-associated depression patients without psychosis or impulsivity disorders, which display worsened depressive symptoms in the off phases of motor fluctuations [46].

Moreover, exploring the potential of anti-inflammatories on PD-associated depression is crucial for understanding the role of inflammation. In 6-OHDA-lesioned animals, neuroinflammation has been shown to contribute to depressive-like behavior. Notably, piroxicam treatment prevented depressive-like symptoms and increased the hippocampal 5-HT levels in the hippocampus [66]. Ibuprofen also demonstrated beneficial effects on depressive-like behavior by modulating oxidative stress and neurodegeneration in a PD model induced by rotenone [67]. Similar results were obtained in TLR4 knock-out mice subjected to MPTP [68]. In addition to anti-inflammatory approaches, ketamine, an N-methyl-D-aspartate (NMDA) receptor antagonist approved as an antidepressant, has emerged as a promising option in the treatment of PD-associated depression. Ketamine treatment counteracts short-term social memory impairment and depressive-like and anhedonia-like behaviors without worsening motor performance in animals subjected to 6-OHDA [69,70].

Importantly, in humans, a randomized, double-blind, placebo-controlled clinical trial in phase II is evaluating the effectiveness of ketamine in treating depression in patients with PD [71]. Nevertheless, to the best of our knowledge, there is a lack of randomized controlled trials for anti-inflammatory drugs in PD-associated depression. At present, a phase II, randomized, placebo-controlled study has investigated the effects of azathioprine, an immunosuppressive medication, in modifying PD. The underlying hypothesis was that modulation of the peripheral immune system may have disease-modifying effects [72]. Another ongoing study examining exosomes in PD (EXPAND) is focused on characterizing mitochondria-derived vesicles as biomarkers in PD patients, seeking to elucidate the interconnection between mitochondrial dysfunction, systemic inflammation, and PD progression [73]. Furthermore, a cohort of patients with PD in Spain, the COPPADIS-2015 study, is seeking to understand the progression of PD, integrating clinical assessments, serum biomarkers, genetic studies, and neuroimaging, which can provide insights into the relationship between inflammation and depression in PD [74]. However, it is crucial to note that it is unclear whether these clinical studies will analyze the association between inflammatory markers and depression in PD.

## 5. Discussion

Overall, this literature reviewed emphasizes the intricate relationship between noradrenaline, 5-HT, and dopamine on PD-associated depression. Of note, the presence of brain inflammation in PD, which modulates and is regulated by noradrenergic, serotonergic, as well as dopaminergic systems, highlights a putative key mechanism for depressive symptom exacerbation. Also, there is a temporal relation between non-dopaminergic dysfunctions in the RN and LC, depression, and inflammation markers, all of them being components of early stages of PD.

However, the main challenge remains. Due to limitations of imaging techniques and standard biomarkers, PD diagnosis is still late. In addition, the current studies have mainly focused on the pharmacological tools for therapeutic approaches, restricting some conclusions about the elucidation of the pathophysiological mechanisms of PD-associated depression. Consequently, data about the in vivo integrity of the RN and LC, neuroinflammation, microglia activation, and their correlation with non-motor symptoms—including depression—both in humans undiagnosed with PD and early-phase PD, are scarce or absent.

**Figure 1:**
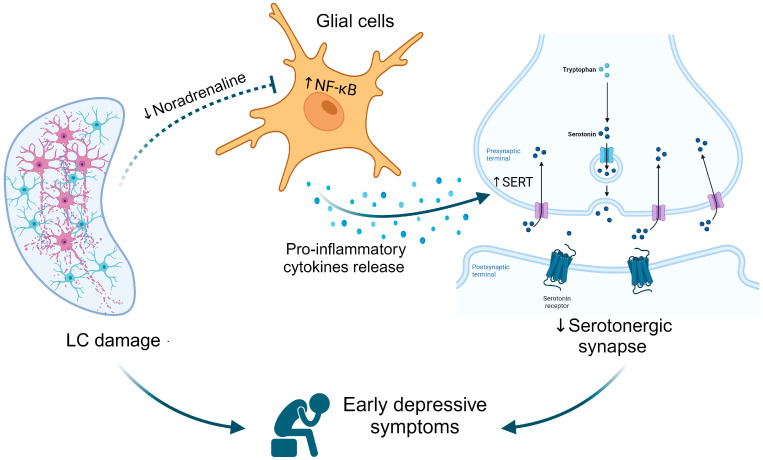
Locus coeruleus (LC) damage culminates in a reduced anti-inflammatory action of noradrenaline on the glial cells resulting in pro-inflammatory cytokine release, which induces a serotonergic impairment by increasing the serotonin uptake. Together, noradrenergic and serotonergic deficits may result in early depressive symptoms observed in Parkinson’s Disease. NF-κB = nuclear transcription factor nuclear factor kappa B; SERT = serotonin transporter.

Thus, although it is hard to determine whether there is a cause–consequence link between these neurotransmitter impairments, inflammation, and PD depression, we hypothesize an intrinsic relationship among them based on: (I) the fact that inflammation is a major factor in PD progression and depression, (II) the anti-inflammatory role of noradrenaline, and (III) Braak’s staging. As illustrated in Figure 1, LC damage is a precedent event (stages 1–2) that induces a central noradrenaline deficit, reducing the anti-inflammatory control of the glial cells. Consequently, pro-inflammatory cytokines, such as TNF-α and IL-1β, are released from activated astrocytes and microglia. Pro-inflammatory cytokines stimulate 5-HT reuptake and/or degradation, impairing the serotonergic pathway. Together, noradrenergic and serotonergic impairments could support the early depressive symptoms in PD patients, which could worsen/shift in the presence of dopaminergic neurodegeneration (stages 3–4).

Of highlight, we herein sought an integrative view of early PD dysfunctions reported in all the monoaminergic neurotransmitter systems and their connections with inflammation because we believe that PD is a multisystemic disorder. In this sense, understanding the influence of each monoaminergic system on PD-associated depression allowed us to rationalize a drug discovery, aiming to more assertively test therapeutic interventions, like multitarget drugs. Moreover, novel imaging techniques and biochemical tools tend to elucidate both the onset and early stages of PD, allowing us—in the near future—to treat PD patients still in the premotor phase. This approach probably will be more effective in stabilizing or slowing PD progression.

However, it is worth highlighting that PD develops and progresses differently among patients; the speed of functional abnormalities on the serotonergic, noradrenergic, or even dopaminergic pathways can also vary and cause the distinct intensity of depressive symptoms in each one. Thus, more studies are needed to correlate individual symptoms, as part of a depressive state or not, with 5-HT/noradrenaline damage.

## 6. Conclusions

In conclusion, we provided an integrative view of monoaminergic neurotransmitter systems in PD, with a focus on early functional abnormalities in noradrenergic and serotonergic pathways and their relationship with the inflammatory process. Importantly, we developed a hypothesis for the early depressive symptoms associated with PD, in which LC–noradrenergic system impairment exacerbates pro-inflammatory mediator release culminating in increased 5-HT uptake and, consequently, monoaminergic neurotransmission deficits.

## Figures and Tables

**Table 1:** Summary of the evidence about the involvement of the serotonergic and noradrenergic pathways on the pathophysiological mechanisms of depression associated with Parkinson’s disease in humans.

Reference	Neurotransmitter Systems	Subjects (n)	Methodology	Main Findings
Mayeux et al., 1984 [47]	Dopaminergic, noradrenergic, and serotonergic	In total, 16 depressed, 25 non-depressed PD patients, and 15 healthy controls	Measurement of 5-hydroxyindoleacetic acid (5-HIAA), homovanillic acid (HVA), and 3-methoxy-4-hydroxyphenylglycol (MHPG) in the cerebrospinal fluid (CSF)	5-HIAA concentration, the main serotonin metabolite, was reduced in the CSF of PD patients with major depression and dysthymic disorder, as compared with that in non-depressed PD patients or controls. PD patients with major depression showed the greatest reduction in 5-HIAA levels in the CSF. The concentrations of HVA and MHPG in the CSF were not linked to the presence of depression.
Berg et al., 1999 [48]	Serotonergic	In total, 20 depressed and 11 non-depressed PD patients	Magnetic resonance imaging (MRI) and transcranial sonography (TCS)	Signal intensity reduction and T2 relaxation time of pontomesencephalic midline structures, which comprise the raphe nuclei (RN), in depressed PD patients as compared to those in non-depressed PD patients. No correlation was found between MRI signals or TCS echogenicity and motor symptoms or depression severity.
Remy et al., 2005 [49]	Dopaminergic and noradrenergic	In total, 8 depressed and 12 non-depressed PD patients	Positron emission tomography (PET) imaging	Depressed PD patients exhibited lower binding in the NET and DAT compared to those patients without depression, especially in the locus coeruleus (LC), anterior cingulate cortex, thalamus, amygdala, and ventral striatum. Anxiety severity in PD patients was found to be inversely correlated with the tracer binding in most of these regions, whereas apathy showed a specific inverse correlation in the ventral striatum.
Frisina et al., 2009 [50]	Dopaminergic, noradrenergic, and serotonergic	In total, 11 depressed and 9 non-depressed PD patients	Post-mortem pathological and immunohistochemical analysis	Pathological features were higher in the LC and dorsal vagus nerve of depressed PD patients as compared to those in non-depressed ones. Gliosis was more prevalent among depressed PD patients. No significant differences were identified in terms of gliosis and neuronal loss in the SN and RN of depressed and non-depressed PD patients.
Ballanger et al., 2012 [51]	Serotonergic	In total, 4 depressed, 8 non-depressed PD patients, and 7 healthy controls	PET imaging	Depressed PD patients demonstrated a reduction in the 5-HT_1A_ receptor density as compared to non-depressed PD patients in the left hippocampus, right insula, left superior temporal cortex, and orbitofrontal cortex. Relative to healthy controls, non-depressed PD patients showed a bilateral decrease in the 5-HT_1A_ receptor density in the inferior frontal cortex, right ventral striatum, and insula. In turn, depressed PD patients showed a specific 5-HT_1A_ receptor decrease in the left dorsal anterior cingulate, orbitofrontal and temporal cortices, and right hippocampus.
Qamhawi et al., 2015 [52]	Serotonergic	In total, 345 early-onset PD patients, 56 putative PD patients without evidence of dopamine deficit, and 185 healthy controls	Single photon emission computed tomography (SPECT)	PD patients showed reduced availability of SERT in the raphe nuclei as compared to healthy controls and putative PD individuals without evidence of dopaminergic deficit. PD patients with resting tremors demonstrated an even more reduced availability of the SERT in the RN and less severe striatal dopaminergic deficits as compared to PD patients with akinetic–rigid syndromes and without tremors at rest. SERT availability in the RN was associated with resting tremor severity but not non-motor symptoms, including depression.
Maillet et al., 2016 [53]	Dopaminergic and serotonergic	In total, 15 Apathetic PD patients and 15 non-apathetic PD patients	PET imaging	Apathetic PD patients had higher depression and anxiety scores compared to non-apathetic PD ones. Non-apathetic PD patients showed mainly dopaminergic denervation in several regions, whereas those with apathy exhibited widespread dopaminergic and serotonergic degeneration in the striatum and thalamus. Specific dopaminergic disruption was reported in the SN–ventral tegmental area complex and specific serotonergic changes were found in the insula, orbitofrontal, and subgenual anterior cingulate cortices. Apathy severity was mainly correlated to specific serotonergic lesions in the right anterior area of the caudate nucleus and the orbitofrontal cortex.

DAT: dopamine transporter; NET: noradrenaline transporter; PD: Parkinson’s Disease; SERT: serotonin transporter; SN: substantia nigra.
